# Self-stigma in suicide prevention of mental health professionals

**DOI:** 10.1093/eurpub/ckac129.530

**Published:** 2022-10-25

**Authors:** S Roškar, D Kralj, K Andriessen, K Krysinska, M Vinko, A Podlesek

**Affiliations:** 1 Public Health, National Institute of Public Health, Ljubljana, Slovenia; 2 Centre for Mental Health, University of Melbourne, Melbourne, Australia; 3 Department of Psychology, University of Ljubljana, Ljubljana, Slovenia

## Abstract

**Introduction:**

Stigma is one of the main factors hindering help-seeking, which can have debilitating effects on mental health and even lead to suicidality. Stigma can affect the general public but also mental health professionals. In this study we examined mental-illness and help-seeking self-stigma as well as public stigma of suicidal behavior among suicidologists.

**Methods:**

Invitation to participate in the study was sent to 518 member of the Internat.l Assoc. for Suicide Prevention. 89 participants (55 female, 34 male; 17% response rate) completed the survey. We gathered sociodemographic data, data on personal history of mental illness and suicidal behavior and on different types of stigma. We hypothesized that help-seeking self-stigma is predicted by sociodemographic attributes and personal history of mental illness and that self-stigma related to mental illness and suicide act as mediators.

**Results:**

Personal experience with mental illness predicted mental illness self-stigma. There was no significant predictive value of other variables (age, gender, years working in suicidology) for self-stigma of mental illness and suicide behavior. Both types of self-stigma (mental illness and suicide behavior) were correlated. Mental-illness self-stigma was shown to be a stronger predictor of help-seeking self-stigma than self-stigma of suicide behavior, though the effect did not reach statistical significance. Self-stigma of suicide behavior showed no independent contribution to help-seeking self-stigma.

**Conclusions:**

Mental healthcare professionals represent a particularly vulnerable group for developing mental health issues and suicidality. However, due to fear of being perceived less competent by colleagues and the public, they often disguise their mental struggles and are reluctant to seek help. These pilot findings warrant further research to better understand self-stigma and its impact on help-seeking behavior in order to prevent suicidality in this population.

